# Supplementary figures and images for: Nanoscopical Analysis Reveals an Orderly Arrangement of the Presynaptic Scaffold Protein Bassoon at the Golgi-Apparatus
Source: Front Mol Neurosci. 2021 Nov 5;14:744034. doi: 10.3389/fnmol.2021.744034 (PMC8632625; doi:10.3389/fnmol.2021.744034)

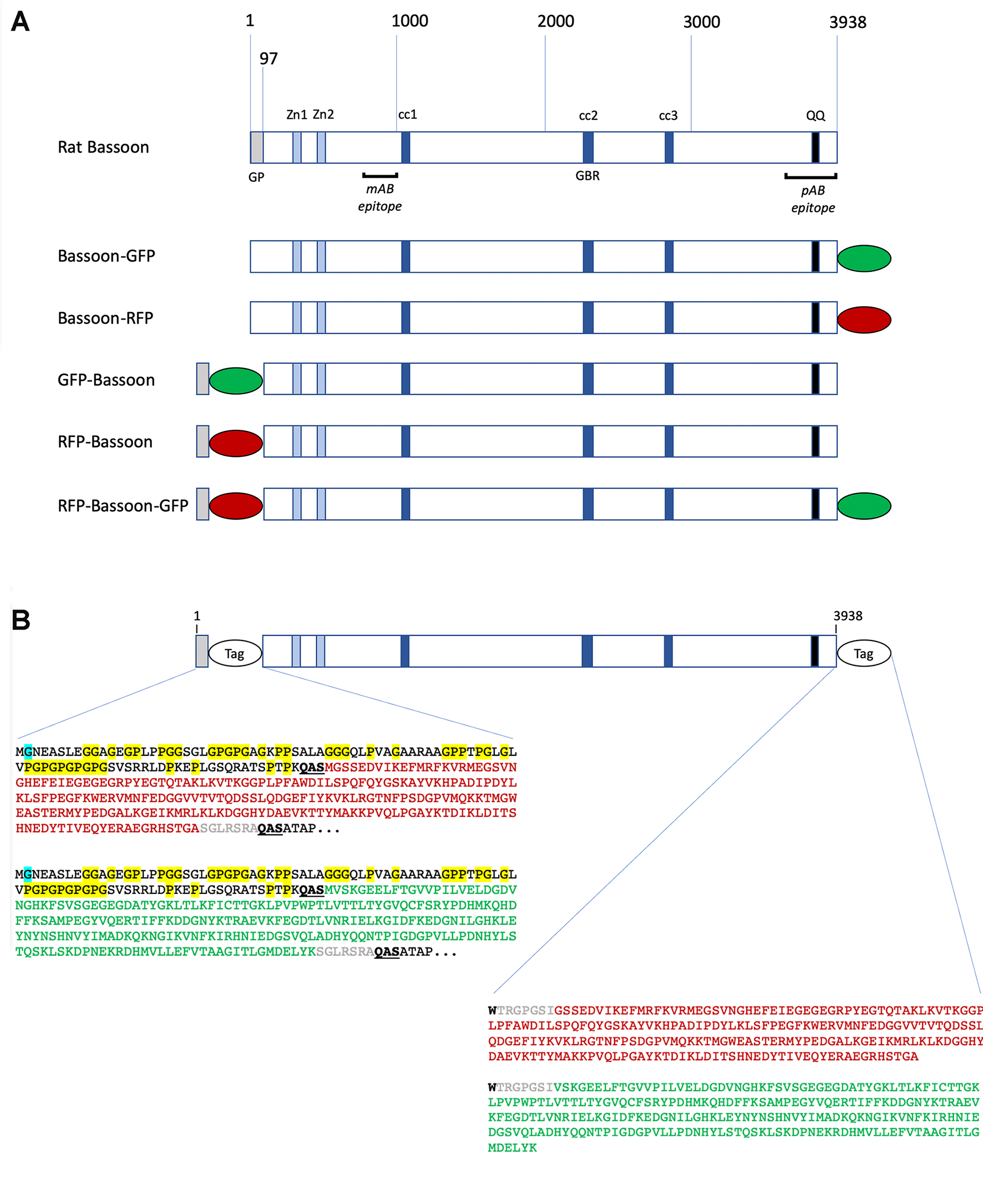

Supplement: Supplementary Figure 1 — (A) Schematic of new Bassoon full-length constructs. Numbers indicate amino acid positions of rat Bassoon. GP indicates the glycine- and proline-rich region comprised of amino acids 1–97; Zn1 and Zn2 indicate zinc finger domains; cc1, cc2, and cc3 are coiled-coil domains; cc2 contains a Golgi-binding region (GBR) of Bassoon; QQ indicates the location of glutamine repeats. The regions-containing epitopes for commercially available antibodies used or discussed in our study are indicated as “mAB” for monoclonal antibody and “pAB” for polyclonal antibody. Green ovals represent mGFP, red ovals represent mRFP. (B) Amino acid sequences of regions containing tags in the novel constructs. Red font: mRFP; green font: mGFP; gray font: spacers; black font: rat Bassoon sequences. The c-terminal tags (either mRFP or mGFP) were placed downstream of the sequence WTRGPSI, where W (bold font) is amino acid 3938 of rat Bassoon. The intramolecular tags (either mRFP or mGFP) were placed downstream of serine 97 of rat Bassoon. The constructs were designed in such a way that Q95, A96, and S97 (QAS, indicated in bold font and underlined) were repeated downstream of the tags. G2, which is part of a predicted N-myristoylation consensus site, is highlighted in blue. Glycine and proline residues located within the first 97 amino acids of Bassoon are highlighted in yellow to indicate their frequent occurrence in this region. [file Image_1.TIF]

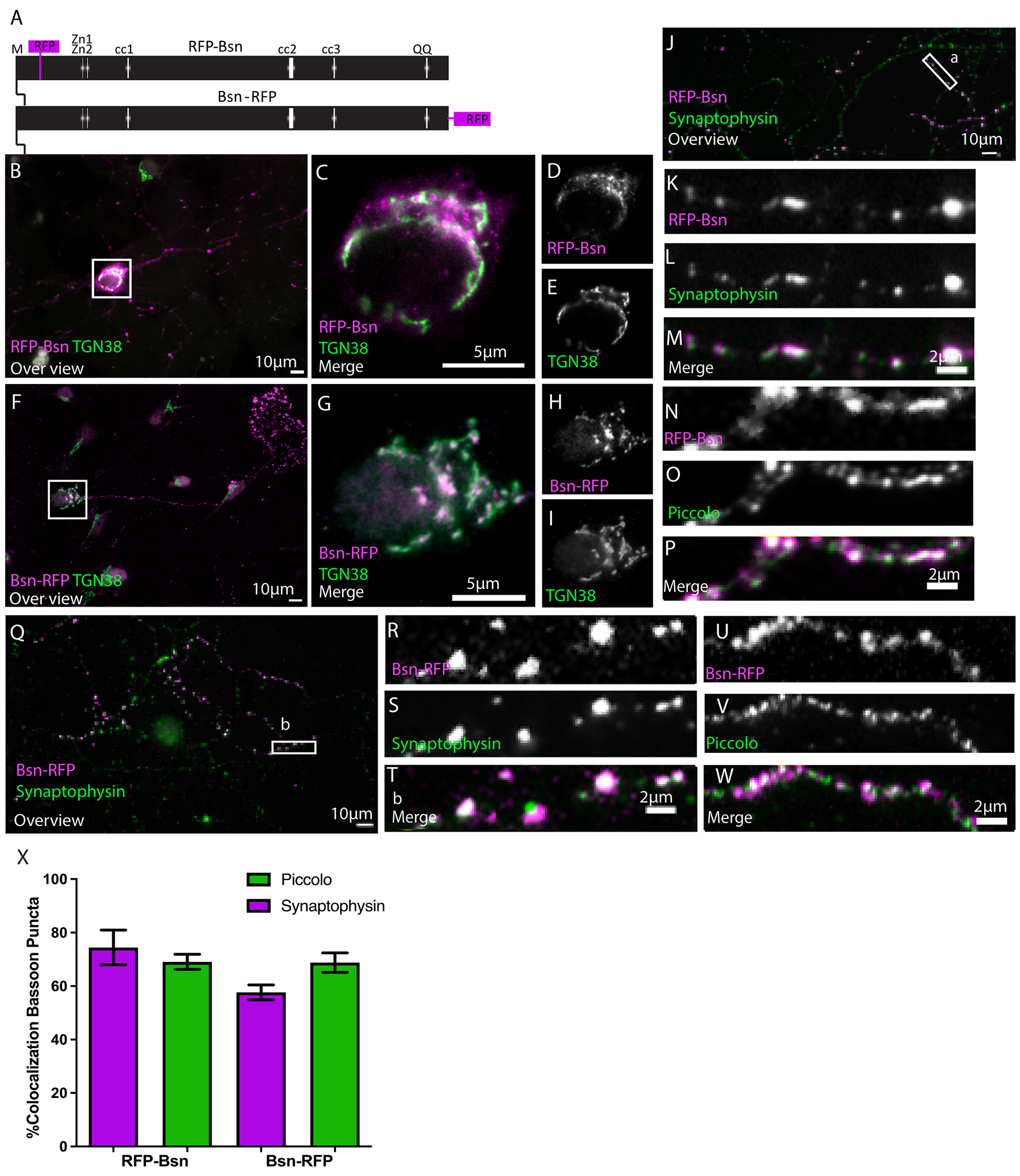

Supplement: Supplementary Figure 2 — Full-length RFP-tagged Bassoon constructs localize at the TGN in young neurons, traffick to synaptic sites, and are incorporated into the insoluble AZ scaffold of mature neurons. Panel (A) is a schematic diagram of full-length Bassoon sequence compared with the sequence of mRFP-tagged Bassoon constructs, where M stands for N-myristoylation sequence, Zn1 and Zn2 are the two zinc finger domains, and cc1, cc2, and cc3 are the three predicted coiled-coil regions. Immunostained DIV5-7 (B–E,F–I) and DIV14 (J–P,Q–W) hippocampal neurons transfected with RFP-tagged Bassoon constructs, post a DIV3 lipofectamine transfection, are co-stained with the TGN38 (B–H), synaptophysin (J–L,Q–S), and piccolo (M–O,T–V). Panels (I,P) represent 40× over views of the transfections, and a and b represent the zooms of their white square ROIs, respectively. Neurons were fixed in cold methanol prior to PFA fixation to quench the RFP autofluorescence. N = 10 transfected neurons/condition imaged (B–I). (X) Is the Bassoon colocalization quantification for panels (J–W); data are represented as mean ± SEM, N = 32 (RFP-BSN-Piccolo), 13 (BSN-RFP-Piccolo), 34 (RFP-BSN-Synaptophysin), 14 (BSN-RFP-Synaptophysin), images analyzed. N = 2–3 separate transfected neuronal cultures/independent experiments (B–W). Scale bars 10 μm (B,F,Q, J), 5 μm (C–G), and 2 μm (K–P,R–W). [file Image_2.TIF]

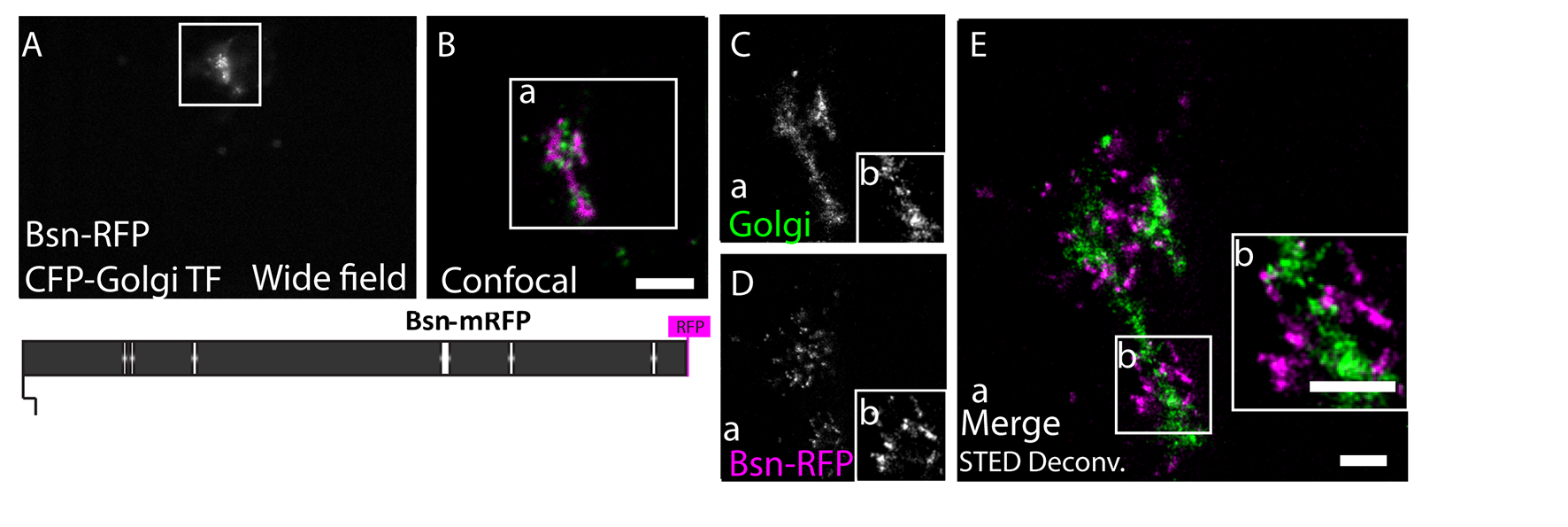

Supplement: Supplementary Figure 3 — Full-length C–terminally tagged Bassoon does not localize to the trans-Golgi sub-compartment labeled by CFP-Golgi. DIV7 hippocampal neurons were transfected with CFP-Golgi (a trans-Golgi sub-compartment maker) and full-length single-tagged Bsn- mRFP construct, and immunostained using GFP and/or RFP nanobodies against tagged constructs and from (A–E). Two-color STED images of Golgi sub-compartment marker CFP-Golgi and RFP-Bsn constructs (C–E). (A) shows wide field overview of transfected constructs, (B) the confocal zooms of the soma, inset a reflects the single channels and merged full STED deconvolved (Deconv.) images of (C–E), while inset b represents the zooms of STED images. N = 10 images of transfected neuronal somas imaged as technical replicates N = 2 separate neuronal cultures/independent experiments. Scale bars 4 μm (B) and 1 μm (E). [file Image_3.TIF]

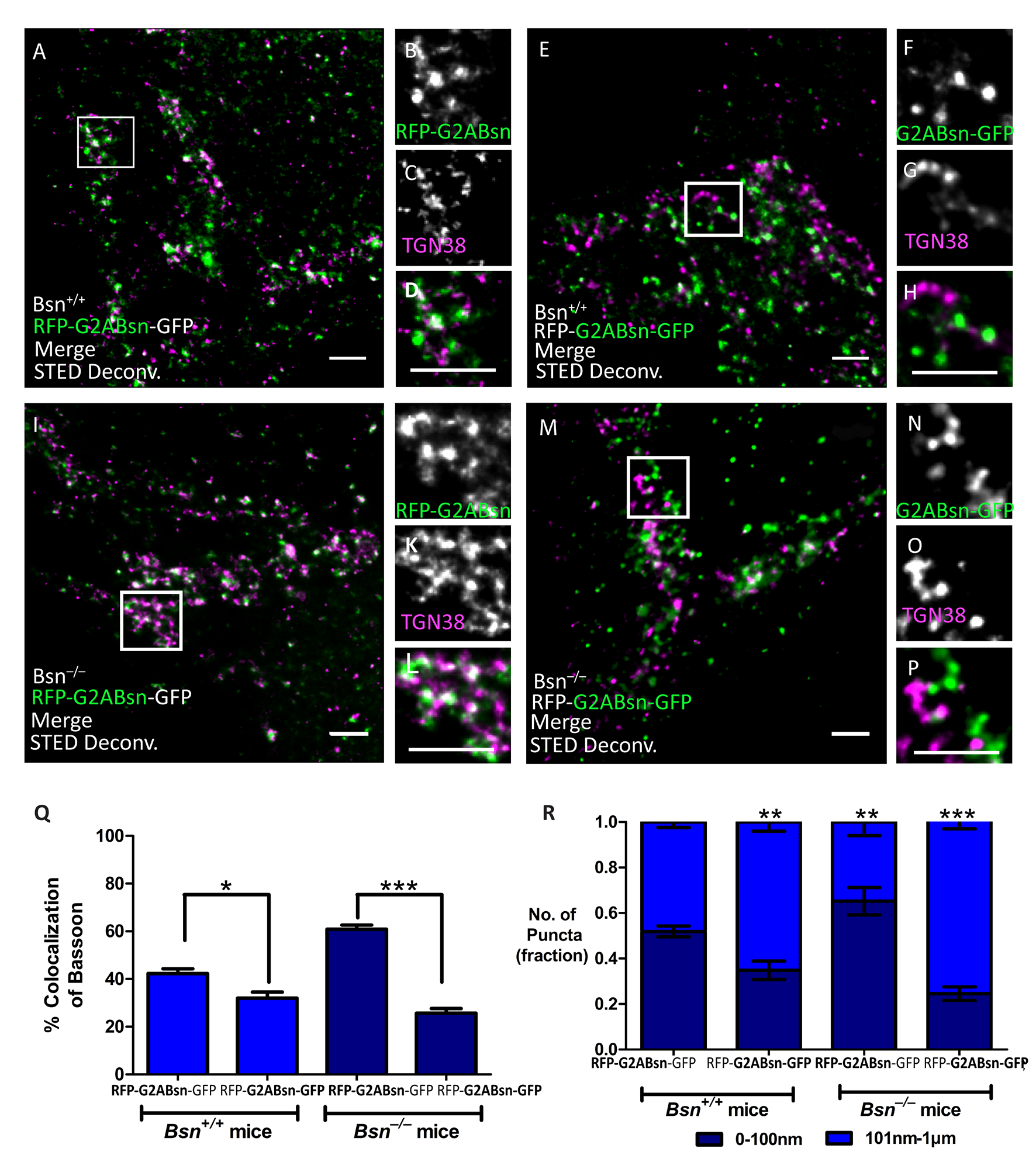

Supplement: Supplementary Figure 4 — Orientation of G2A-mRFP-Bsn-mEGFP myristoyl mutant construct in endogenous Bassoon-free Bsn–/– knockout mice and their Bsn+/+ wild-type littermates. DIV7 Bsn+/+ (A–H) and Bsn–/– (I–M) sandwich hippocampal cultures were transfected with G2A-mRFP-Bsn-mEGFP. Two-color STED images with their respective insets are shown for the N– (A–D,I–L) and C– (E–H,M–P) termini of the myristoyl mutant construct, respectively. Immunostaining was performed using RFP-nanobody-Atto594 or GFP-nanobody-Atto647 and a TGN38 marker. Graphs (Q,R) were represented as mean ± SD, N = 4 transfected neuronal soma image/condition. Data are represented as mean ± SEM. N = 2 knockout and two wild-type animals. Quantified and statistically tested with a Tukey’s multiple comparisons test for amount of colocalization and signal distributions, respectively, ∗p ≤ 0.05,∗∗p ≤ 0.01, and ∗∗∗p ≤ 0.001. Scale bars 1 μm (A–P). [file Image_4.TIF]

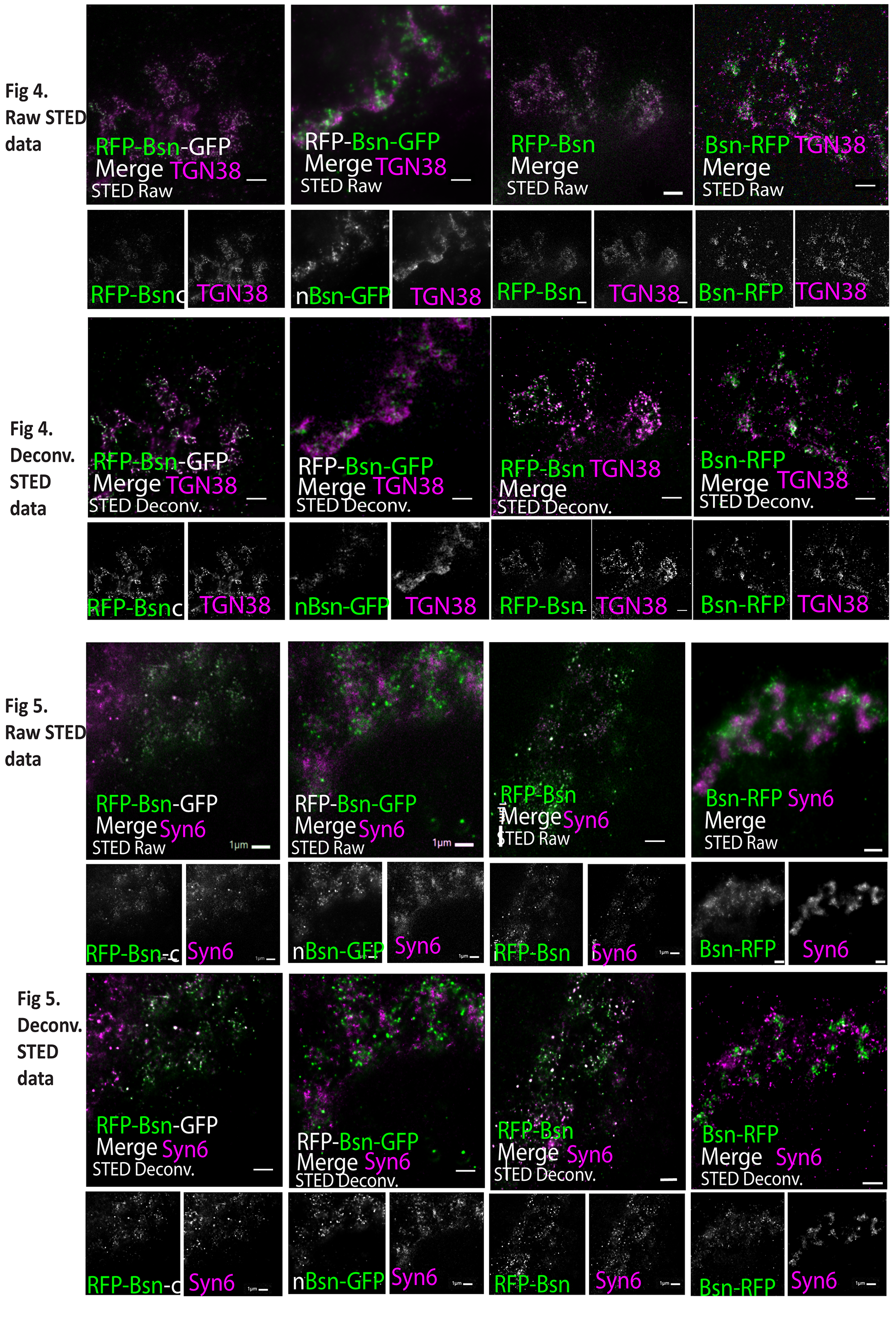

Supplement: Supplementary Figure 5 — Raw and deconvolved images of Figure 4 and Figure 5 transfected full-length Bassoon constructs. Transfections were performed in DIV7 hippocampal cultured neurons. The Bassoon signals are visualized by GFP/RFP nanobodies and co-stained with the TGN38/Syn6 marker. Raw STED data with individual channels and merged overviews of each image and the following row show the same images after Richardson Lucy deconvolution. N = 10 transfected neurons/condition imaged and N = 2 separate transfected neuronal cultures/independent experiments. Scale Bar = 1 μm. [file Image_5.PNG]
